# Season, Vegetation Proximity and Building Age Shape the Indoor Fungal Communities’ Composition at City-Scale

**DOI:** 10.3390/jof8101045

**Published:** 2022-10-03

**Authors:** Hélène Niculita-Hirzel, Pascal Wild, Alexandre H. Hirzel

**Affiliations:** 1 Department Work, Health & Environment, Center for Primary Care and Public Health (Unisanté), University of Lausanne, Route de la Corniche 2, CH-1066 Epalinges-Lausanne, Switzerland; 2 Computer Science Center, Amphimax Building, Quartier Sorge, University of Lausanne, CH-1015 Lausanne, Switzerland

**Keywords:** indoor air quality, urban mycobiome, built environment, metabarcoding, culture

## Abstract

Exposure to particular microbiome compositions in the built environment can affect human health and well-being. Identifying the drivers of these indoor microbial assemblages is key to controlling the microbiota of the built environment. In the present study, we used culture and metabarcoding of the fungal Internal Transcribed Spacer ribosomal RNA region to assess whether small-scale variation in the built environment influences the diversity, composition and structure of indoor air fungal communities between a heating and an unheated season. Passive dust collectors were used to collect airborne fungi from 259 dwellings representative of three major building periods and five building environments in one city—Lausanne (Vaud, Switzerland)—over a heating and an unheated period. A homogenous population (one or two people with an average age of 75 years) inhabited the households. Geographic information systems were used to assess detailed site characteristics (altitude, proximity to forest, fields and parks, proximity to the lake, and density of buildings and roads) for each building. Our analysis indicated that season was the factor that explained most of the variation in colonies forming unit (CFU) concentration and indoor mycobiome composition, followed by the period of building construction. Fungal assemblages were more diverse during the heating season than during the unheated season. Buildings with effective insulation had distinct mycobiome compositions from those built before 1975 — regardless of whether they were constructed with pre-1945 technology and materials or 1945 — 1974 ones. The urban landscape—as a whole—was a significant predictor of cultivable *Penicillium* load—the closer the building was to the lake, the higher the *Penicillium* load—but not of fungal community composition. Nevertheless, the relative abundance of eleven fungal taxa detected by metabarcoding decreased significantly with the urbanization gradient. When urban landscape descriptors were analyzed separately, the explanatory power of proximity to vegetation in shaping fungal assemblages become significant, indicating that land cover type had an influence on fungal community structure that was obscured by the effects of building age and sampling season. In conclusion, indoor mycobiomes are strongly modulated by season, and their assemblages are shaped by the effectiveness of building insulation, but are weakly influenced by the urban landscape.

## 1. Introduction

In urban societies, buildings are the main places where people live, work and entertain [1]. Therefore, the quality of indoor air conditions the respiratory health of the inhabitants. One regular indoor pollutant incriminated in the induction of respiratory health problems in young children [2,3] (as in adults) [4] when present at an abnormally high concentration, are the bioaerosols, in particular those emitted by fungal species growing on built humid surfaces (following water leaks in the envelope, plumbing failures, or condensation). Nevertheless, while low-diversity mycobiomes in dwellings were strongly and significantly associated with an increased risk of later asthma development [5,6], exposure to high-diversity mycobiomes in the first years of life has been associated with a protective effect against allergy [7,8,9]. Thus, it has been proposed that the loss of diversity results in a so-called “dysbiosis” of the mycobiome associated with the built environment—a state that describes the altered composition of this microbial community, which has a cascading impact on the immune system and offers an advantage for the emergence and outbreak of pathogens [10].

The association between exposure to a particular mycobiome profile and adverse health outcomes [11] has prompted research into the key factors that determine the composition and diversity of the indoor mycobiome. Such research has been facilitated in the last years by the increased knowledge of the diversity and composition of mycobiomes through the use of next generation DNA sequencing (NGS) technology on the microbial barcode markers, such as nuclear ribosomal internal transcribed spacer (ITS) for fungi [12]. The NGS has proven to be a useful tool for characterizing microbial diversity in a variety of indoor environments at sufficient depth to highlight the importance of various sources of microorganisms in driving airborne mycobiome diversity. 

The most studied source of fungi in the built environment is that of building materials contaminated by molds. A wide range of fungi have been identified to proliferate on building materials containing cellulose—such as wood or wallpaper—when moisture levels are high enough to allow their growth [13,14,15,16,17,18]. Fungal spores and hyphal fragments are released from these surfaces based on the life cycle of the fungus and the air velocity at the surface of the material [19,20]. In addition, in bathrooms, fungal particles can be aerosolized from contaminated surfaces when water droplets splashes onto them in showers [21]. The concentration of fungi released in aerosols from moldy surfaces was reported to be high enough to distinguish mycobiomes of moldy or water damaged buildings from those of “healthy” dwellings [20,22]. Interestingly, one good predictor of indoor mold growth prevalence is the period of building construction [23,24,25]. Indeed, certain construction periods have an increasing risk for dampness problems due to the building materials used, the lack of ventilation and the level of insulation that favors moisture accumulation [26,27]. Thus, the residential buildings constructed in the thirties often present unfavorable thermal profiles in the walls or floor, while those constructed after the eighties are too airtight [28,29]. In addition, occupancy density and occupant behavior also play a role in shaping indoor mycobiomes by modulating air temperature and humidity levels, and by contributing with their own microbiome (e.g., pets, plants [30], fungi associated with human skin [31]). Airborne fungi can be released into the built environment not only from contaminated water-damaged materials [22], but also from other building characteristics, such as air-conditioning (HVAC) systems [32]. 

The composition of the indoor mycobiome relies not only on indoor sources but also on the composition of the outdoor mycobiome, which might contribute significantly to the indoor mycobiome [33]. The variation in fungal composition of outdoor air varies dynamically across time and space at continental [34], global [35] and even at city [31] scales. This variation in composition is related to the season [36], and to the nature of the surrounding landscapes (ocean, agricultural soil, forest etc.) from which local meteorological conditions (especially wind direction and speed) control the release and dispersal of fungal spores [37,38,39,40,41]. In particular, the type and density of vegetation in urban and rural areas condition airborne microorganisms [25,42], with each plant species hosting distinct microbial communities [40,43]. Nevertheless, the infiltration rate of outdoor fungi indoors depends on the airtightness of the building envelope and the frequency of window opening by the occupants [20,44], with both indoor and outdoor temperatures having a significant impact on the window opening behavior of occupants [45,46]. While the spatiotemporal variation of the microbiome has been explored in urban outdoor air, the influence of urban landscape diversity on the indoor mycobiome during heating and unheated periods has been little documented [31]. Understanding how the urban landscape relates to diversity and function of the indoor mycobiome is necessary to understand the mycobiome ecology, and to be able to guide urban development in ways that utilize ecological functions to enhance human health and well-being. 

The aim of the study was to reveal the influence of the urban landscape on indoor mycobiomes across time and space in a city with a wide variation in altitude, surrounding landscape (e.g., lake, forest), urbanization level, and with a long-term construction history, Lausanne. Three construction periods have been identified according to the materials used, and the ventilation and isolation requirements of the building envelope [29]. Therefore, dwellings built at the same period are expected to share more taxa than those constructed in other periods. Given that Lausanne is in a temperate region and that outdoor temperature influences the occupants’ behavior to open windows, we hypothesize that indoor mycobiomes fluctuate between the heating and unheated periods. In the unheated period with optimal outdoor fungal growth conditions, we expect the indoor air mycobiome to be influenced by the local outdoor environment (altitude, proximity to forest, fields and parks, proximity to the lake and road density). To test these hypotheses, Electrostatic Dust Collectors (EDC) were installed in 259 dwellings inhabited by a homogenous population (one or two people with an average age of 75 years) during the heating and unheated periods. The location of the dwellings represents the heterogeneity of the built environment of Lausanne. Fungi present in the samples were surveyed through culturing and DNA metabarcoding analyses of the rDNA ITS2 region.

## 2. Materials and Methods

### 2.1. Experimental Design

**Figure 1:**
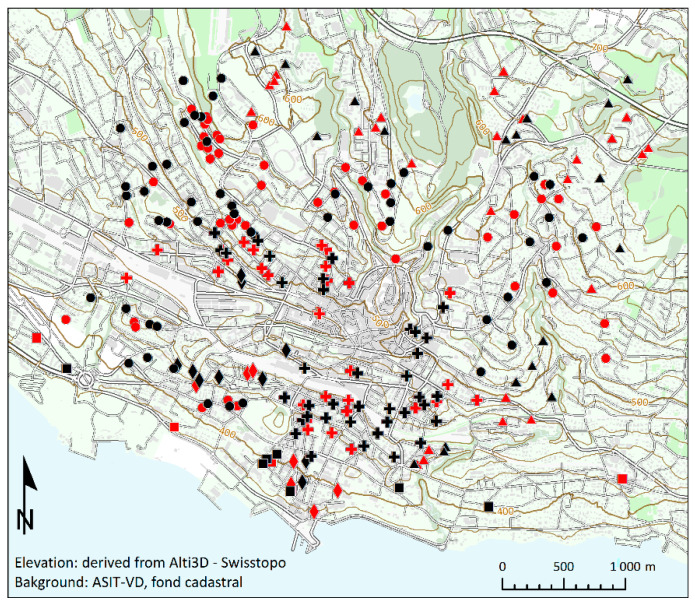
Geographical location of the buildings for which the mycobiome was characterized, in black for those for which molecular and cultivable data were obtained, and in red for those for which only the culture provided results. Their belonging to an environmental typology is indicated by a triangle for peri-urban cluster, by a circle for the suburban cluster, by a plus for the historic downtown cluster, by a diamond for the lakefront downtown, and by a square for the lakefront. Elevation lines every 100 m and 20 m are indicated with thick and thin brown lines, respectively.

To include dwellings in Lausanne with a homogenous population, the study was proposed at the follow-up consultation of the Lc65+ cohort [47]. The Lc65+ cohort was composed of people aged 71–80 years in the year of the study, living alone or in couples. Of the 417 participants who come to the consultation, 391 were eligible (they lived in Lausanne and did not moved or intend to move from the same address), and 287 agreed to participate in the study (87 from early June to late August and 197 from early September to late December). They all lived in Lausanne in buildings with natural ventilation. Each participant filled in a short self-reported questionnaire with dwelling characteristics (number of persons living in the apartment, presence of visible mold, orientation of the sampled room, floor number), and left the consultation with one electrostatic dust collector (EDC) [48] as well as instructions on how to install it in their main living room between 1.20 and 1.60 m above the floor, and an envelope to return the EDC to the lab. The EDCs consist of commercially available electrostatic wipes (Apta Captizz, Apta, France), set in a plastic case, which the participant opens to collect dust passively. Wipes were first sterilized in an autoclave (124 °C for 30 min) and stuck on a disinfected polypropylene case (DVD single-slim case, BECO GmbH & Co. KG, Arnsberg, Germany) with white glue (UHU^®^, Bühl, Germany). The system has been previously validated for its effectiveness in trapping the overall fungal diversity present in aerosols [49,50], this includes the sampling time, as well as the methods for fungal DNA extraction and amplification of the indoor fungal species [40,51]. After 10 weeks, participants returned the EDC by mail. The EDCs returned between September and late October were considered representative of the unheated period, while those returned between early November and late February were representative of the heating period. The location of the dwellings was confirmed as representative of the different urban environments of Lausanne (Figure 1). 

### 2.2. Mycobiomes Characterization

A total of 259 EDC were returned by mail. Once in the lab, each EDC wipe was placed in a plastic bag with 20 mL of a solution of Tween 80 to 0.1% (Merck^®^, Darmstadt, Germany) and washed for 5 min in a Stomacher ™ (AES^®^, Combourg, France). An aliquot of the harvested liquid was kept for culture, while the rest was centrifuged for 30 min at 8500 *g* and the liquid discarded. The recovered pellet was mechanically disrupted for 2 min at 30 Hz with a TissueLyser (Qiagen, Hombrechtikon, Switzerland) in the first buffer of a FastDNA Spin Kit for Soil (MP Biomedicals, Illkirch, France). The total DNA was extracted according to the manufacturer’s instructions. A two-step PCR approach combined with Illumina’s dual indexing strategy was chosen in order to process all of the samples in parallel. To describe the fungal communities, the internal transcribed spacer 1 (ITS1), the genomic region the most commonly used in metabarcoding studies of fungi, has been chosen. To amplify this barcode, the primer pair selected was the one the most commonly used for this marker: ITS1F and ITS2, to which the following universal tail was added, respectively 5′TCGTCGGCAGCGTCAGATGTGTATAAGAGACAG and 5′GTCTCGTGGGCTCGGAGATGTGTATAAGAGACAG. Each DNA sample was amplified in triplicate in 20 μL reaction volumes containing 10 μL DNA, 4 μL of 5× Phusion HF Buffer, 0.4 μL of dNTP Mixture (10 mM each), 1 μL of each NGS primer (10 μM each), 0.6 μL of DMSO, and 0.6 U of Phusion High-Fidelity DNA Polymerase (NEB, Ipswich, MA, USA). DNA amplification conditions in the Biometra PCR thermal cycler were 98 °C for 2 min, then 35 cycles of each, 98 °C for 30 s, 55 °C for 30 s, and 72 °C for 1 min, and a final elongation step of 10 min at 72 °C. By that stage, 131 samples were successfully amplified and passed the DNA quality control by electrophoresis on a 3% agarose gel. A correlation between the number of CFUs in culture and DNA extraction efficiency was observed (unsuccessfully amplified Mean ± SD = 20 ± 18 CFU·day^−1^, N = 128; successfully amplified Mean ± SD = 25 ± 20 CFU·day^−1^, N = 131; *p* = 0.01). The amplicons—of a size of ~450 bp—were purified with Agencourt AMPure XP (Beckman Coulter, Brea, CA, USA), resuspended in 40 μL of 10 mM TrisCl pH 8 and quantified using the Quant-IT^TM^ PicoGreen^TM^ dsDNA reagent and kit (Life Technologies corporation, Oregon, OR, USA). Libraries were prepared from the resulting amplicons using the Illumina MiSeq Reagent Kit v2 and sequenced on a Illumina MiSeq platform (by Microsynth, Balgach, Switzerland) to generate 250 bp paired-end reads. 

To determine the cultivable fraction, 250 μL of the harvested suspension were spread in replicates on plates with dichloran-glycerol culture medium (DG18) (Oxoid PO5088A, Thermo Fisher Diagnostics AG, Pratteln, Switzerland) and placed in an incubator at 25 °C for five days. All plates were checked daily for fungal colony growth and total and phenotype specific fungal colony forming units (CFUs) were counted. The different phenotypes were identified at the genus level by macroscopic observation of the growth characteristics of the colonies and the microscopic observation of conidiophores, branching patterns of conidiophores and conidiogenous cells morphology [52,53]. Each CFU concentration was normalized per day of sampling by multiplying the number of CFUs by the volume of the EDC wash solution and dividing by the number of days sampled.

### 2.3. Bioinformatics

Forward and reverse reads were merged, trimmed, and quality filtered using the following criteria: no differences to primer and identifier, overlap 20, average quality > 28, minimum sequence length 100 bp, homopolymer length < 9. Tags and primer sequences were removed. The resulting 11,493,895 amplicon sequence variants were further clustered into operational taxonomic units (OTUs) using the UCLUST algorithm in QIIME v.1.7.0 [54] at a 97% similarity cut-off. The taxonomic assignment of sequences were done with a QIIME preformatted database of UNITE [55] and the international nucleotide sequence databases NCBI, EMBL and DDBJ, by using BLASTN algorithm for the taxonomic assignment over UCLUST. Chimeras, singletons, and contaminants were discarded. The final dataset contained 493,005 OTUs accounting for 9,396,225 reads. The number of reads per sample varied from 4571 to 357,813 with a mean value of 69,090 and a median value of 44,513 reads. The number of OTUs per sample varied from 191 to 11,099. The dataset was rarefied to an even depth of 9000 reads per sample. This led to one sample being discarded prior to downstream statistical analyses. A table with the distribution of identified OTUs through samples has been generated. As the taxonomical accuracy for the ITS1 sequence amplified with ITS1F/ITS2 primers can be resolved at genus but not species level [56], a second table was generated with the OTUs pooled at the genus level.

### 2.4. Environmental Variables

Complete data on the year of construction of the building and on the built environment were extracted based on the addresses of the participants of the Lc65+ cohort that still live in Lausanne (1881). The buildings’ construction year, the heating system, the heating agent and the living surface were retrieved from the ‘Registre Cantonal des Bâtiments’. Building environmental variables—altitude (Alti3D), proximity to forest, fields and parks (cadastral data), proximity to the lake (SwissTLM3D, density of buildings and roads (SwissTLM3D)—were retrieved from Swisstopo, the Swiss Federal Office of Topography, databases with the models indicated in brackets. Geographic information systems were used to associate the site characteristics (altitude, proximity to forest, fields and parks, and proximity to the lake, and density of buildings and roads) for each building. Lausanne’s climate is classified as warm-summer (“Dfb”) by the Köppen Climate Classification system. The heating period covers the months of November to March, while the unheated period is from April to October. 

### 2.5. Statistical Analysis

The building environment variables retained were confirmed to be independent with a principal component analysis. Therefore, a typology of built environments was generated using an agglomerative hierarchical clustering method based on the Euclidean distance. This algorithm uses the farthest pair of observations between two groups to determine the proximity between two groups. The mycobiome was analyzed in respect to the location of buildings. The buildings represented the five typological environments (named peri-urban, suburban, historic downtown, lakefront downtown and lakefront) and the three construction periods defined by building characteristics and insulation efficiency (before 1944: cement slab with metal beams, hollow body in clay or wooden floor, solid wall, window with single glass; between 1945 and 1974: concrete slab, simple or compound wall without insulation, window with two single glass panes without insulation; between 1975 and 2014: slab/floor with insulation, composite wall with insulation, window with double insulating glass; [29]) for both the heating and unheated periods. In order to identify the principal factors that influence the fungal communities’ composition, we used Analysis of Similarity (AnoSim) with 1000 permutations, unless otherwise stated, and principal coordinates analysis (PCoA = classical multidimensional scaling) on all pair wise Bray-Curtis to display the dissimilarities between samples graphically. The alpha diversity of fungal assemblages was estimated by the Shannon (H′) index and the beta diversity by Bray-Curtis dissimilarity distance. Genera-specific analyses were carried out using logistic regression for their frequencies (presence absence in samples) and the adapted non-parametric test— either the Kruskal-Wallis rank test or the Wilcoxon rank-sum test—for the taxa relative abundance in samples. Statistics were performed in R version 3.6.2. [57] through RStudio (version 1.3.959) or with Stata software version 17.0 (StataCorp LLC, College Station, TX, USA).

## 3. Results

### 3.1. Building Environments

The clustering algorithm of the 1881 addresses of the entire Lc65+ cohort identified five distinct clusters of urban landscapes across Lausanne, based on elevation, density of roads and buildings, proximity to green spaces and to the lake. These clusters have been named the peri-urban, suburban, historic downtown, lakefront downtown, and lakefront clusters respectively for ease of tracking on the Figure 1. A total of 259 sampled dwellings, evenly distributed across these five urban landscapes and representative of the different construction periods, are shown in Table 1. Cultivable fungi were detected in all samples collected (259 dwellings), and mycobiomes were characterized in 131 of them (Figure 1). All dwellings had district heating.

### 3.2. Cultivable Fraction of Fungi & Environment

The concentration in cultivable fungi was significantly higher during the heating than during the unheated period (*p* = 0.000, Table 2). *Penicillium* and *Aspergillus* were the most frequently detected genera in cultures, accounting for the majority of colonies forming units (CFUs) in 21% and 7% of the dwellings, respectively—48 dwellings sampled during the heating period and nine during the unheated period for *Penicillium*; nine during the heating period and 11 during the unheated period for *Aspergillus*. While the concentration of *Aspergillus* CFUs in dwelling dust did not vary significantly between heated and unheated seasons or between urban landscape clusters, the concentration of *Penicillium* CFUs was higher during the heating than during the unheated season (*p* = 0.020, Table 2) In particular, the concentration of *Penicillium* CFUs was higher in dwellings constructed after 1975 (*p* = 0.034, Table 2), and correlated with the urban landscape gradient (Coef. = 2.92 *p* = 0.009)—with the lowest concentration in the peri-urban cluster, and the highest in the lakefront cluster. Fungal contamination was reported in 14 dwellings (5%), mostly during the heating period (N = 13). No correlation was observed between homeowner reporting of visible mold and concentration of cultivable fungi (*p* = 0.839). Moreover, while the concentration of cultivable fungi was higher during the heating season in dwellings built after 1975 (*p* =0.008, Table 2), no significant association between visible mold and construction period was observed.

### 3.3. Factors Driving the Diversity of Indoor Fungal Community

Taxonomical identification of the retained OTUs revealed 248 distinct fungal genera across the indoor environments. Statistical analysis confirmed that fungal richness varied widely between the unheated and heating period. Fungal diversity was significantly higher in dwellings sampled during the heating period than during the unheated period (Shannon index: *p* = 0.0006; fungal richness: *p* = 0.0000; Table 2). No association of fungal diversity or richness with urban landscape or construction period was detected. Fungal richness was inversely correlated with the relative abundance of the most abundant genera independently of the sampling season. Note that the relative abundance of the most abundant taxa in the samples was significantly higher in dwellings with visible mold (50.4 ± 29.3 versus 29.4 ± 13.9, *p* = 0.0004). Interestingly, only *Aspergillus* showed a higher relative abundance in mold contaminated dwellings compared to those with no visible molds declared (*p* = 0.0003).

### 3.4. Factors Structuring the Composition of Indoor Fungal Community

**Figure 2:**
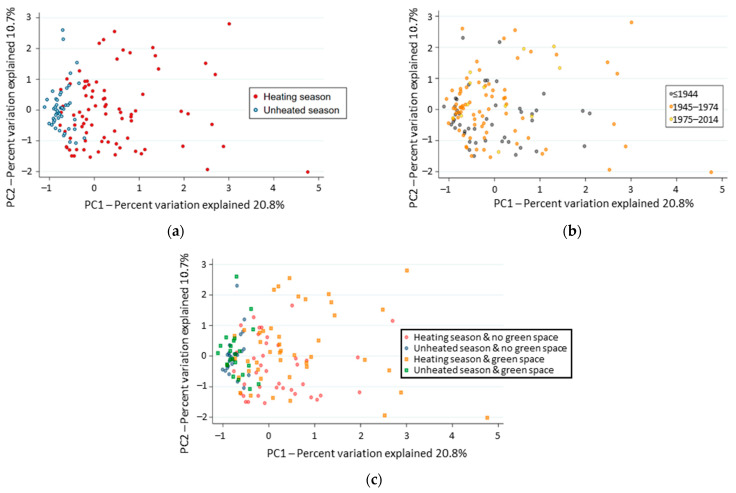
Visualization of the similarity in genus composition of fungal communities across the studied buildings. Each point represents the fungal community in a building, such that those that are closer together share more identified genera in common than those farther apart. Fungal community composition and relative abundance of genera tend to cluster according to season (**a**), period of building construction (**b**), or proximity to green space in the surroundings (**c**).

Two principal factors, with Eigenvalues of 20.8 and 10.7, structured the composition of fungal communities when OTUs were regrouped at the genus level. The first factor clustered the fungal communities according to sampling period (*p* = 0.000; Figure 2a), although the second factor score regrouped the fungal communities according to the building construction period (*p* = 0.037; Figure 2b). Significant post-hoc differences were observed between the composition of the fungal communities sampled in the most recent buildings (1975–2014) and those built before 1944 (*p* = 0.016). No correlation was observed between the two principal factor scores and urban landscape clusters. However, the second factor score differed according to the proximity of green space (*p* = 0.013; Figure 2c), in particular during the unheated period (*p* = 0.020). The presence of a dominant phenotype in culture explained little of the beta diversity of the fungal communities (3.3% of the variation; *p* = 0.047), while the report of visible molds was associated with a disperse distribution of the beta diversity across samples in the PCoA plot. The mycobiomes collected from dwellings located at different floors or with different cardinal orientation were similar (*p* = 0.342). 

### 3.5. Fungal Indicator Taxa associated with Seasons

**Figure 3:**
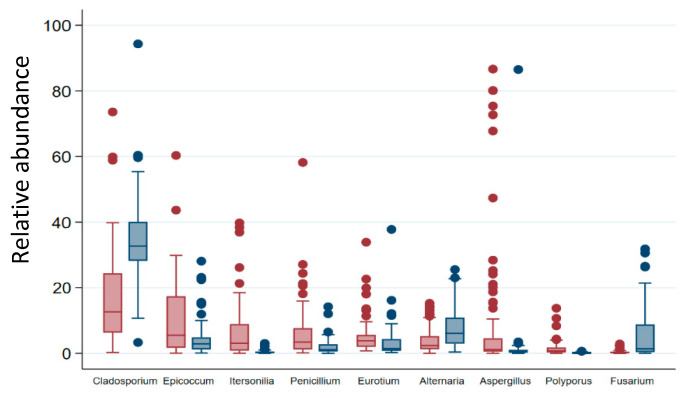
Boxplots showing distribution in relative abundance of the nine most abundant and frequent genera across samples. The values observed during the heating period are shown in red, and those during the unheated period are shown in blue. The whiskers in a box-and-whisker plot are the adjacent values that correspond to the highest value not greater than p75 + 3/2 IQR and the lowest value not less than p25 − 3/2 IQR, where IQR is the inter-quartile range, the box covers the values between the first and third quartile, and the line in the box marks the median value.

**Figure 4:**
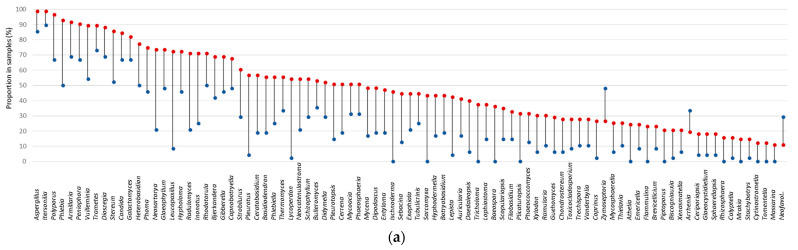

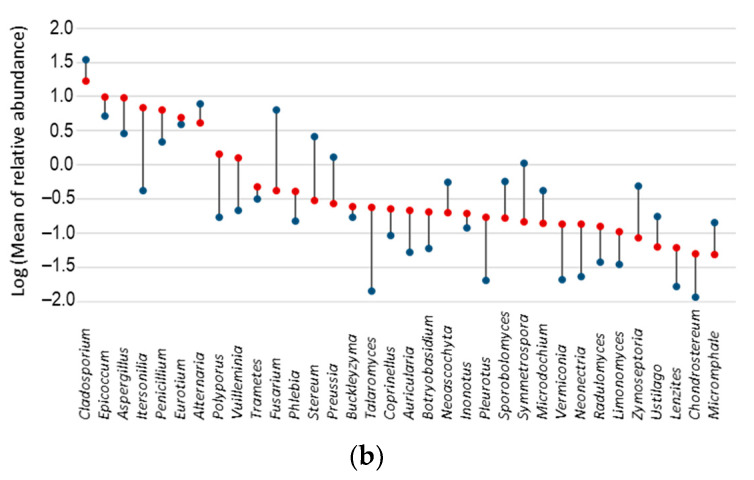
Visualization of the proportion (**a**) and log transformed mean relative abundance (**b**) of indoor fungal genera that differed significantly between the unheated and heating periods (*p* < 0.05). Values observed during the heating period are shown in red, and those observed during the unheated period are in blue.

The most abundant taxa detected in samples by next generation sequencing—*Cladosporium*, *Penicillium*, *Alternaria*, *Epicoccum*, *Eurotium*, *Itersonilia*, *Aspergillus* and *Fusarium*—were also the most frequently detected in samples (in 100%, 99%, 99%, 98%, 98%, 95%, 94%, and 92% of samples, respectively). However, the range of their relative abundance in samples was significantly different between the unheated and heating periods: *Aspergillus*, *Epicoccum*, *Itersonilia*, *Penicillium* and *Eurotium* were among those with the highest relative abundance in samples during the heating period, while *Cladosporium*, *Alternaria* and *Fusarium* accounted for those with a greater number of reads in samples collected during the unheated season (Figure 3). A total of 109 of the 248 taxa identified showed significant differences in their frequency or/and relative abundance in the samples between the unheated and heating periods. Of these, 89 taxa showed a significant difference in frequency between the unheated and heating periods, of which only three were more frequent during the unheated than during the heating period (Figure 4a). Thirty-four taxa showed a significant difference in their relative abundance between the unheated and heating periods, of which 22 taxa were more abundant during the heating period and 12 were more abundant during the unheated period (Figure 4b). Fourteen taxa showed a significant difference between the unheated and heating periods in both frequency (presence/absence) and relative abundance. 

### 3.6. Fungal Indicator Taxa associated with the Construction Period of the Building

**Figure 5:**
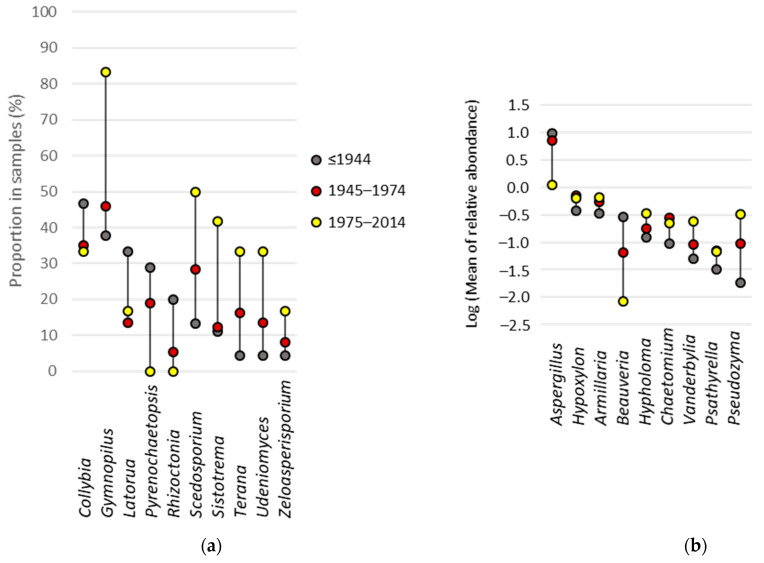
Visualization of the proportion (**a**) and log transformed mean relative abundance (**b**) of indoor fungal genera that differed significantly between buildings constructed at different periods (*p* < 0.05). Shown in grey are taxa observed in buildings constructed before 1944, in red are those observed in buildings constructed between 1945 and 1974, and in yellow are taxa observed in buildings constructed between 1975 and 2014.

Among the 19 taxa identified as indicators of building age categories, 10 varied in the frequency with which they have been detected in buildings of different ages and nine in their relative abundance. The frequency of these taxa in samples increased with building age for three taxa and decreased with building age for six taxa (Figure 5a); only one taxon, *Latorua*, did not show such association. In addition, the relative abundance increased with building age for *Aspergillus* and *Beauveria* (Figure 5b), and decreased with building age for *Armillaria*, *Hypholoma*, *Vanderbylia* and *Pseudozyma*. Two of those, *Armillaria* and *Hypholoma,* were also among the genera with the highest prevalence in the samples (83% and 63%, respectively).

### 3.7. Fungal Indicator Taxa associated with the Building Environment 

**Figure 6:**
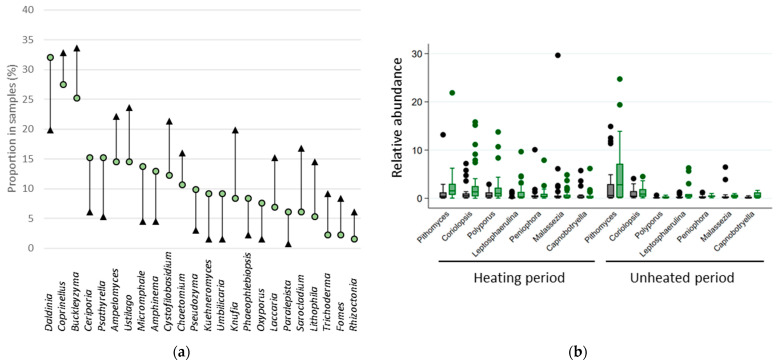

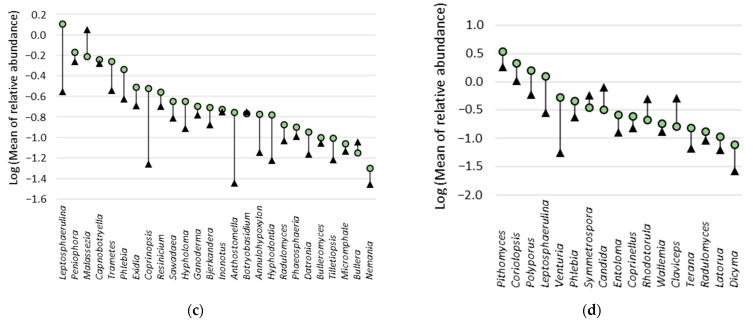
Visualization of the proportion (**a**) and mean log transformed relative abundance (**c**,**d**) of indoor fungal genera that differed significantly between buildings with and without green space in the surroundings (*p* < 0.05)—(**c**) during the unheated period (**d**) during the heating period. The distribution of relative abundance among samples is illustrated for few taxa in (**b**). In green, values for buildings with green space in the surroundings, and in black, values for buildings without green space in the surroundings.

The urban landscape gradient—from peri-urban to lakefront—was correlated with a decrease in relative abundance of 11 taxa: *Coriolopsis* (Coef. = −0.509 *p* = 0.026), *Daldinia* (Coef. = −0.188 *p* = 0.030), *Datronia* (Coef. = −0.032 *p* = 0.038), *Hypoxylon* (Coef. = −0.132 *p* = 0.015), *Microdochium* (Coef. = −0.070 *p* = 0.024), *Panus* (Coef. = −0.029 *p* = 0.047), *Phlebia* (Coef. = −0.085 *p* = 0.022), *Polyporus* (Coef. = −0.375 *p* = 0.035), *Shizophyllom* (Coef. = −0.062 *p* = 0.050), *Trametes* (Coef. = −0.028 *p* = 0.028), and *Vuilleminia* (Coef. = −10.324 *p* = 0.019). Nevertheless, the component of the building environment that most affected the composition of the fungal community was the proximity to vegetation (presence of vegetation patches at 100 m). Proximity to vegetation was significantly correlated with the frequency in samples of 24 taxa (Figure 6a), the relative abundance of 26 taxa during the unheated period (Figure 6c), and 17 taxa during the heating period (Figure 6d). Interestingly, while the presence of green space in the dwellings’ surroundings was associated with higher frequency for 45% of taxa, it was associated with higher relative abundance of fungi for 92% of taxa during the unheated period and 82% during the heating period (Figure 6). The distribution of relative abundance among samples is illustrated for the most abundant taxa in Figure 6b.

## 4. Discussion

In this study, we monitored building-associated fungi by culture and metabarcoding in dwellings along an urban to rural gradient to determine the extent to which season and urban landscape influence fungal assemblages by accounting for the period of building construction. Our results support that within a diverse urban landscape, indoor fungal communities are shaped differently by building characteristics and outdoor factors. Season was associated with distinct values of species richness and load of cultivable fungi. The period of building construction and proximity to green space shaped the fungal community composition as well as the heating period. Moreover, the distance of the building from the lake correlated with the load of *Penicillium* in culture. 

The culturing of environmental samples is the most commonly used method to quantify indoor fungal loads. The present study confirmed that the most common cultivable indoor fungi in a Swiss city are those already reported in cities in neighboring countries—*Penicillium*, *Aspergillus*, and *Cladosporium* [58]. *Penicillium* and *Aspergillus* species are of particular interest because these genera have often been identified as dominant indoor fungi in damp homes, and their concentrations exceed those in outdoor environments [27]. Our results confirmed the seasonal effect on the cultivable fungal load previously described, with higher abundance of *Penicillium* during the heating than during the unheated period [59]. The significantly higher concentration of *Penicillium* in dwellings built after 1975—dwellings that differ from those built before by slab, wall and window insulation—suggests that this seasonal effect could be due to insufficient ventilation; the buildings are too airtight and air renewal is conditioned by the users’ habit of opening the window. In parallel, the higher concentration of *Penicillium* in buildings near the lake suggests a contribution of the relative humidity of outdoor air, which is of 66% during the unheated period and can reach 78% during the heated period [60], to indoor air humidity. Unfortunately, we were unable to assess the contribution of each of these factors by adding season, building construction period and urban landscape clusters in the same model, because the number of sampled buildings was insufficient. Nevertheless, it is known that both the insulation of naturally ventilated buildings and the relative humidity of the air contribute directly and indirectly to availability of water for fungi [61]. Therefore, water availability is generally the limiting factor for fungal growth indoors, as because sufficient nutrients are provided to support fungal growth on dust particles. The increase in the concentration of cultivable *Penicillium* in indoor dust points in this direction, as such an increase has been associated with water damage [58]. However, the lack of correlation between the concentration of fungi detected by culture and visible molds did not allowed us to confirm this link. Such a discrepancy has been so consistently reported that Reboux et al. have concluded that observing the extent of moldy surfaces in a given dwelling is less reliable and less accurate than measuring fungal concentrations in the air for detecting hidden mould [58].

Metabarcoding offers the opportunity to more accurately assess potential exposures resulting from the built environment, as it reveals not only spores, but also the hyphae fragments present in indoor air. Therefore, we characterized the diversity and richness of indoor fungal in a well-represented urban landscape. Fungal diversity and richness were both found to be significantly higher in dwellings during the heating period than during the unheated period. This is in agreement with the results of the previous study of Adams et al. [33], who showed that during the unheated period, outdoor fungi make indoor fungal assemblages more similar to each other, whereas housing specificities are revealed during the heating period. Nevertheless, our data support that season is a greater contributer to mycobiome richness and diversity than the density of green area within a 100 m buffer zone, in contrast to previous reports [25]. Furthermore, while the decrease in diversity during the unheated period has previously been suggested to be related to an influx of high amounts of a few taxa, such as *Cladosporium* and *Alternaria* [25], no such association was observed in our data. Thus, while *Cladosporium*, *Alternaria*, *Fusarium* and *Pithomyces* do account for a large number of reads in samples collected during the unheated season, *Aspergillus*, *Epicoccum*, *Itersonilia*, *Penicillium* and *Eurotium* also account for a large number during the heating season. Regarding the source of these dominant taxa, previous studies have suggested an outdoor origin for some fungi, in particular in the presence of plants, such as for *Cladosporium*, *Alternaria*, *Fusarium* and *Pithomyces*, and an indoor origin for others where moisture damage has been observed in dwellings, such as for *Aspergillus* and *Penicillium* [62,63]. Nevertheless, it cannot be excluded that different sources contribute to the relative abundance of certain taxa. An example is *Cladosporium*. While outdoor air is the source of the majority of *Cladosporium* species found in indoor air (e.g., *C. cladosporioides*, *C. herbarum*) [64,65], moisture damage favors the development on indoor surfaces of other *Cladosporium*, such as *C. sphaerospermum* [64]. Note that our sampling design intentionally removed some sources of variation in the buildings, including the occupancy density and occupants’ behavior, type of heating, type of ventilation, type of houses, etc. Therefore, these factors cannot contribute to the variation in mycobiome composition observed in the study during the heating period.

Building materials and construction methods (e.g., isolation material, ventilation system) have changed over time [29,66]. These differences have been associated with a different composition of fungal community [66,67]. Thus, older properties have been shown to need more maintenance to prevent the ingress of water [68], while dwellings constructed after 1975 are too airtight, which favors the accumulation of humidity in indoor air [69]. Nevertheless, the period of building construction is an indicator that has rarely been considered in recent metabarcoding studies [25]. Our findings support the importance of building age in structuring the indoor mycobiome and the small effect of the built environment, findings that are in agreement with the study by Weikl et al. [25]. Thus, while the age of the building is significantly associated with the composition of fungal communities, the density of buildings and roads in building surroundings has no effect on them. By identifying the indicators’ fungal species, we were able to go a step further, allowing us to see the association between the period of building construction and the relative abundance of *Aspergillus*—a taxon frequently associated with respiratory outcomes. The consistency of this finding is supported by the report of a significant difference in the concentration of *Aspergillus/Penicillium* spores, which was found to be higher in buildings over 90 years old than in properties 31 to 60 years old [27]. The growth of *Aspergillus* on indoor materials is well documented. Depending on the species and the water activity of the substrate, *Aspergillus* can also be a primary, secondary or tertiary colonizer of indoor surfaces. It grows preferentially on concrete or plaster [67].

It has been suggested previously that the geographic location of buildings shapes the composition of fungal communities [31]. We did not find such a strong effect of urban landscape on indoor fungal communities in Lausanne. Only one component of the urban landscape significantly seemed to be associated with a distinct composition of the indoor fungal community, namely the proximity of vegetation. Our findings confirm the importance of the vegetation in a 100 m buffer zone around urban buildings in shaping the composition of the indoor microbiome, as previously proposed by Weikl et al. for an urban area in Munich [25].

Human skin is widely colonized with mycobionts [70], which can be transferred from skin to indoor air. Nevertheless, these taxa have been rarely reported (less than 6% of all sequences or clones) in metabarcoding studies [33,71]. The cause could be that indoor air seems to be the vector rather than the recipient [31]. Therefore, we focus specifically on human associated fungi to detect the impact of the environment on their prevalence and abundance in EBC. Interestingly, we detected five of the genera usually associated with human skin in a majority of indoor environments—*Candida* (78% of samples), *Cryptococcus* (97% of samples), *Debaromyces* (70% of samples), *Malassezia* (92% of samples) and *Rhodotorula* (63% of samples). Two taxa showed a seasonal pattern, with a higher frequency during the heating than during the unheated period: *Candida* (*p* = 0.019, 84% vs. 67%),) and *Rhodotorula* (*p* = 0.016, 71% vs. 50%), suggesting the contribution of the inhabitants’ skin microbiota to the indoor air mycobiome. However, three of them—*Candida* (*p* = 0.03, 0.80 ± 1.5 vs. 0.32 ± 0.44), *Cryptococcus* (*p* = 0.010%; 1.3 ± 1.2 vs. 2.6 ± 4.0) and *Rhodotorula* (*p* = 0.045%; 0.50 ± 0.86 vs. 0.21 ± 0.28)—were more abundant in dwellings without nearby vegetation, suggesting that skin-associated taxa in the indoor air mycobiome may also originate from the outdoor environment, as suggested by Tong et al. [31]. 

## 5. Conclusions

In summary, our study provides evidence that culture and metabarcoding have provided additional and novel information about how buildings and the urban landscape shape indoor fungal communities. For the most studied taxa in indoor air—*Aspergillus* and *Penicillium*—cultivation showed an increase in *Penicillium* spore concentration when the building was constructed after 1975 or when it was closer to the lake, while metabarcoding showed that the relative abundance of *Aspergillus* increased with building age. However, metabarcoding provides more detail on the factors that shape the indoor fungal community composition. For example, we showed that proximity to vegetation has a significant influence on indoor fungal community structure that is masked by the effects of building age and sampling season. Nevertheless, the level of urbanization had limited influence.

## Figures and Tables

**Table 1:** Characteristics of dwellings retained in the present study by comparison to those occupied by seniors of the overall cohort Lc65+.

Construction Period	Building Environment Cluster					Total N (% in Column)
	Periurban	Suburbs	Historical Town Centre	Town Centre near the Lake	Lake Shore	
Seniors buildings in the cohort (N = 1881), N (% in line)						
until 1944	61 (10.0)	169 (28.8)	276 (47.1)	54 (9.2)	26 (4.4)	586 (31.2)
1945–1974	313 (29.1)	519 (48.2)	149 (13.8)	66 (6.1)	30 (2.8)	1077 (57.3)
1975–2014	53(24.3)	130 (59.6)	27 (12.4)	5 (2.3)	3 (1.4)	218 (11.6)
Seniors buildings analyzed by culture (N = 259), N (% in line)						
until 1944	11 (11.6)	29 (30.5)	42 (44.2)	9 (9.5)	4 (4.2)	95 (35.5)
1945–1974	34 (23.3)	69 (47.3)	31 (21.2)	7 (4.8)	5 (3.4)	146 (54.5)
1975–2014	10 (37.0)	13 (48.2)	2 (7.4)	2 (7.4)	0 (0.0)	27 (10.1)
Seniors buildings analyzed by sequencing (N = 131), N (% in line)						
until 1944	4 (8.9)	12 (26.7)	22 (48.9)	6 (13.3)	1 (2.2)	45 (34.3)
1945–1974	15 (20.3)	33 (44.6)	16 (21.6)	4 (5.4)	6 (8.1)	74 (56.5)
1975–2014	2 (16.7)	9 (75.0)	1 (8.3)	0 (0.0)	0 (0.0)	12 (9.6)

**Table 2:** Concentration of cultivable fungi, fungal diversity and richness in samples depending on sampling period and building construction period.

	**Total**	**<1945**	**1945–1974**	**1975–2014**	***p* Value**
Heating period					
Number of samples analyzed by culture	183	65	100	18	
Total CFUs per day, Mean ± SD	26 ± 20	24 ± 17	25 ± 17	40 ± 35	0.008
*Aspergillus* CFUs per day, Mean ± SD	3 ± 12	3 ± 11	3 ± 10	7 ± 23	0.497
*Penicillium* CFUs per day, Mean ± SD	10 ± 21	9 ± 19	9 ± 17	22 ± 37	0.034
Number of samples analyzed by metabarcoding	83	27	48	8	
Shannon index, Mean ± SD	3.5 ± 0.8	3.5 ± 0.9	3.5 ± 0.7	3.6 ± 0.6	0.947
Fungal richness, Mean ± SD	94 ± 34	99 ± 31	91 ± 37	102 ± 29	0.495
Unheated period					
Number of samples analyzed by culture	76	25	43	8	
Total CFUs per day, Mean ± SD	14 ± 15	19 ± 22	12 ± 11	9 ± 5	0.141
*Aspergillus* CFUs per day, Mean ± SD	4 ± 11	6 ± 15	3 ± 7	1 ± 2	0.453
*Penicillium* CFUs per day, Mean ± SD	4 ± 11	7 ± 17	4 ± 6	1 ± 2	0.267
Number of samples analyzed by metabarcoding	48	18	26	4	
Shannon index, Mean ± SD	3.1 ± 0.6	3.1 ± 0.6	3.0 ± 0.7	3.2 ± 0.4	0.910
Fungal richness, Mean ± SD	62 ± 32	60 ± 28	63 ± 33	73 ± 49	0.790

## Data Availability

The data presented in this study are available on request from the corresponding author. The data are not publicly available due their partial use in the present publication.
